# Supplementary material for: Structure and mechanism of a phosphotransferase system glucose transporter
Source: Nat Commun. 2024 Sep 12;15:7992. doi: 10.1038/s41467-024-52100-3 (PMC11393339; doi:10.1038/s41467-024-52100-3)
Supplement: Supplementary file 1 — Supplementary Information [file 41467_2024_52100_MOESM1_ESM.pdf]

Supplementary Information

Structure and mechanism of a phosphotransferase system glucose transporter

Patrick Roth, Jean-Marc Jeckelmann, Inken Fender, Zöhre Ucurum, Thomas Lemmin and Dimitrios Fotiadis*

Institute of Biochemistry and Molecular Medicine, Medical Faculty, University of Bern, Bern, Switzerland

* Corresponding author: dimitrios.fotiadis@unibe.ch

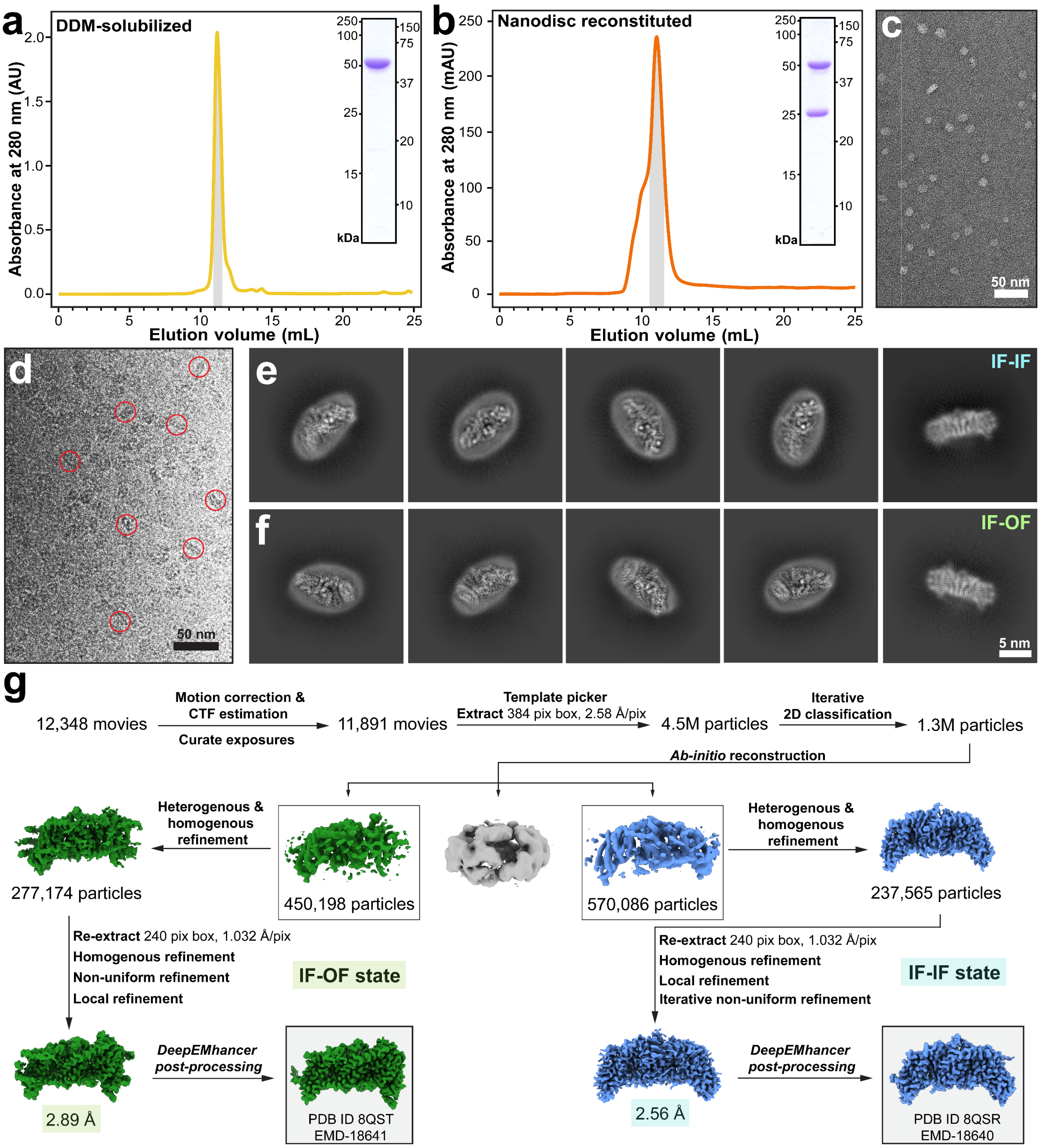

Supplementary Fig. 1: Biochemical and electron microscopical characterization, and cryo-EM structure determination of IICB^{Glc}. (**a**) Size-exclusion chromatography elution profile of *n*-dodecyl-β-D-maltoside (DDM)-purified and (**b**) in lipid nanodiscs-reconstituted IICB^{Glc}, and corresponding Coomassie blue-stained 13.5% SDS-PAGE gels of the indicated peak fractions (in gray). (**c**) Negative-stain EM micrograph of IICB^{Glc} in nanodiscs. (**d**) Representative cryo-EM image with selected particles encircled in red. (**e**) and (**f**) show selected 2D class averages of IICB^{Glc} dimers in nanodiscs in IF (inward-facing)-IF and IF-OF (outward-facing) conformations, respectively. (**g**) Detailed workflow of cryo-EM data processing leading to the final 3D density maps of IICB^{Glc} in nanodiscs in the IF-OF (*left*) and IF-IF (*right*) conformational states. Protein data bank (PDB) and electron microscopy database (EMD) identifiers of final maps are indicated. Abbreviations used: CTF, contrast transfer function; M, millions; pix, pixel(s).

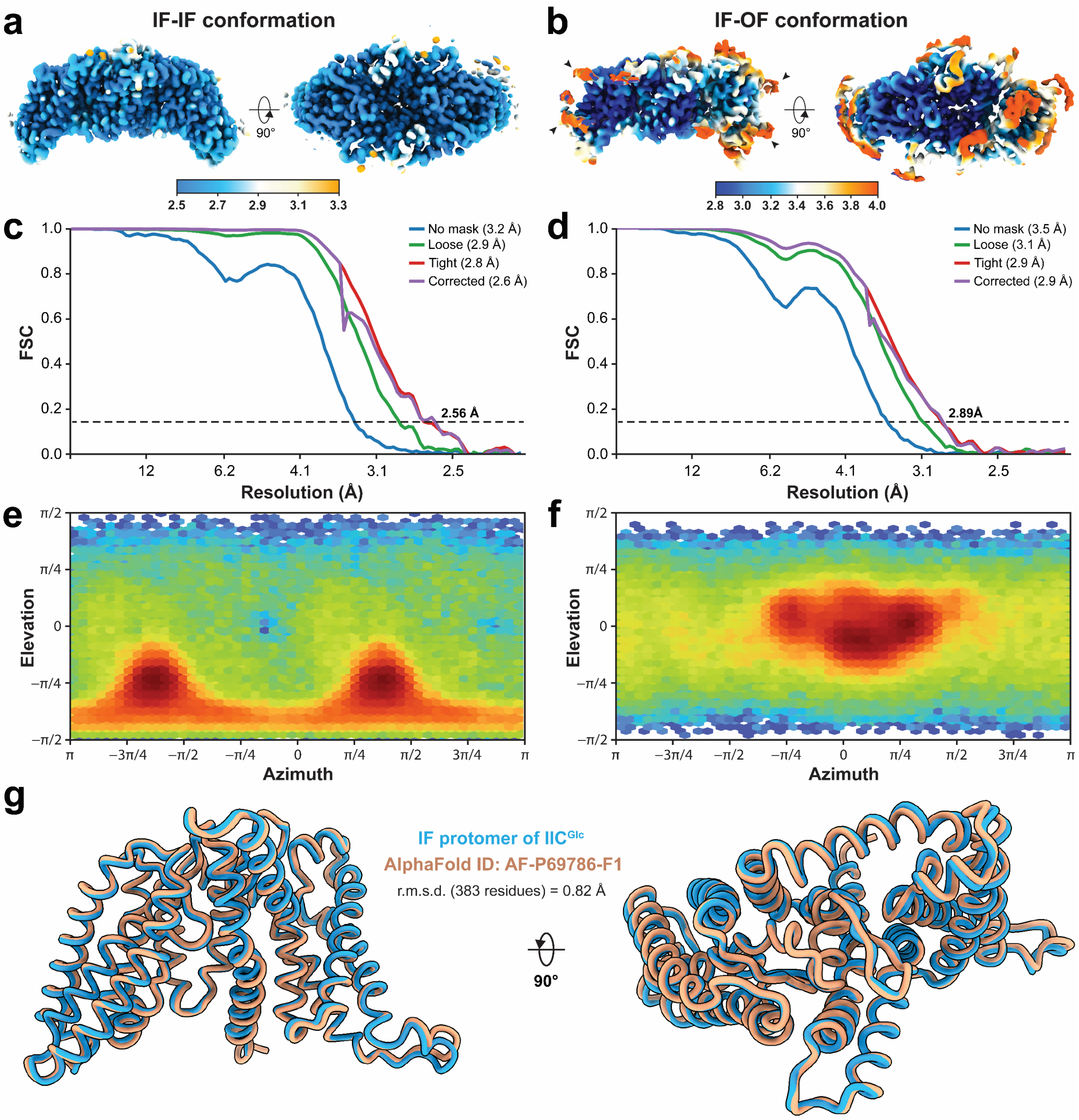

Supplementary Fig. 2: Validation of IF (inward-facing)-IF and IF-OF (outward-facing) IICB^{Glc} dimer cryo-EM structures and comparison with the predicted AlphaFold protomer model. (**a**) and (**b**) show the density maps as side and top views, coloured by local resolution estimates. Numbers in the bars correspond to Å. The low-resolution density around IIC^{Glc} in (b) corresponds mainly to the membrane scaffold protein MSP (arrowheads). (**c**) and (**d**) display the obtained resolutions of the final density using the gold-standard Fourier-shell correlation (FSC) cut-off of 0.143. (**e**) and (**f**) indicate angular distribution plots from cryoSPARC. (**g**) Structural comparison of the experimentally determined IF protomer with the predicted AlphaFold model (AlphaFold database identifier: AF-P69786-F1).

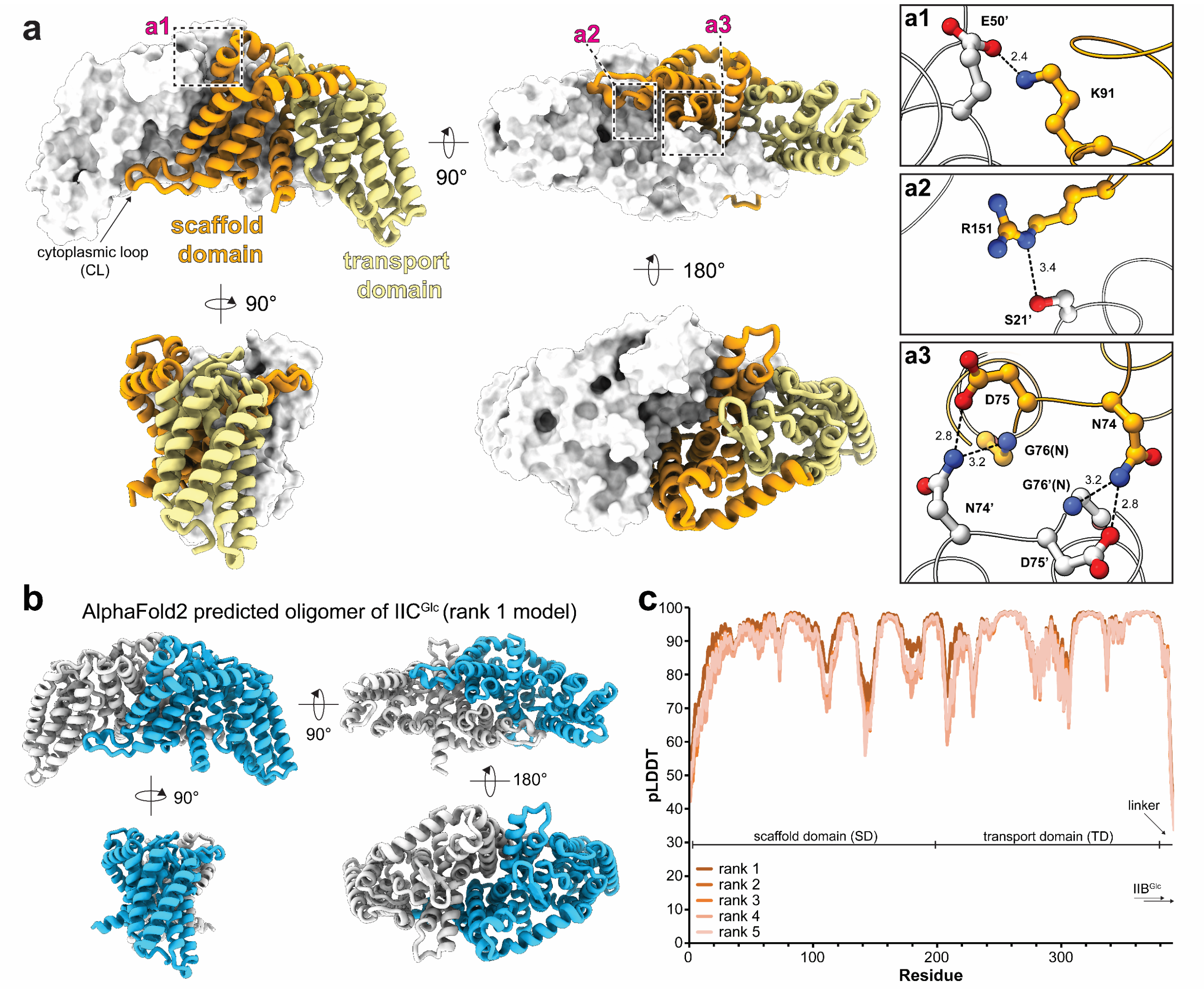

Supplementary Fig. 3: The dimer interface in IIC^{Glc}. (**a**) Structural views of the dimeric assembly in the IF (inward-facing)-IF state. One protomer is shown as ribbon, with the scaffold domain (SD) in orange and transport domain (TD) in yellow. (**a1**), (**a2**) and (**a3**) show the zoomed views of the salt bridge and hydrogen bond networks. Distances are indicated in Å, and the prime symbol indicates amino acid residues of the opposite protomer. (**b**) Predicted IIC^{Glc} dimer (residues 1-386) with AlphaFold2 (rank 1 model, coloured by protomer). (**c**) The per residue confidence score pLDDT (predicted local difference test) of the five top ranked predicted structures. The first four residues of TM1 and the C-terminal linker toward the IIB^{Glc} protein domain are predicted to be disordered (pLDDT<50), and are consistently not visible in the cryo-EM maps.

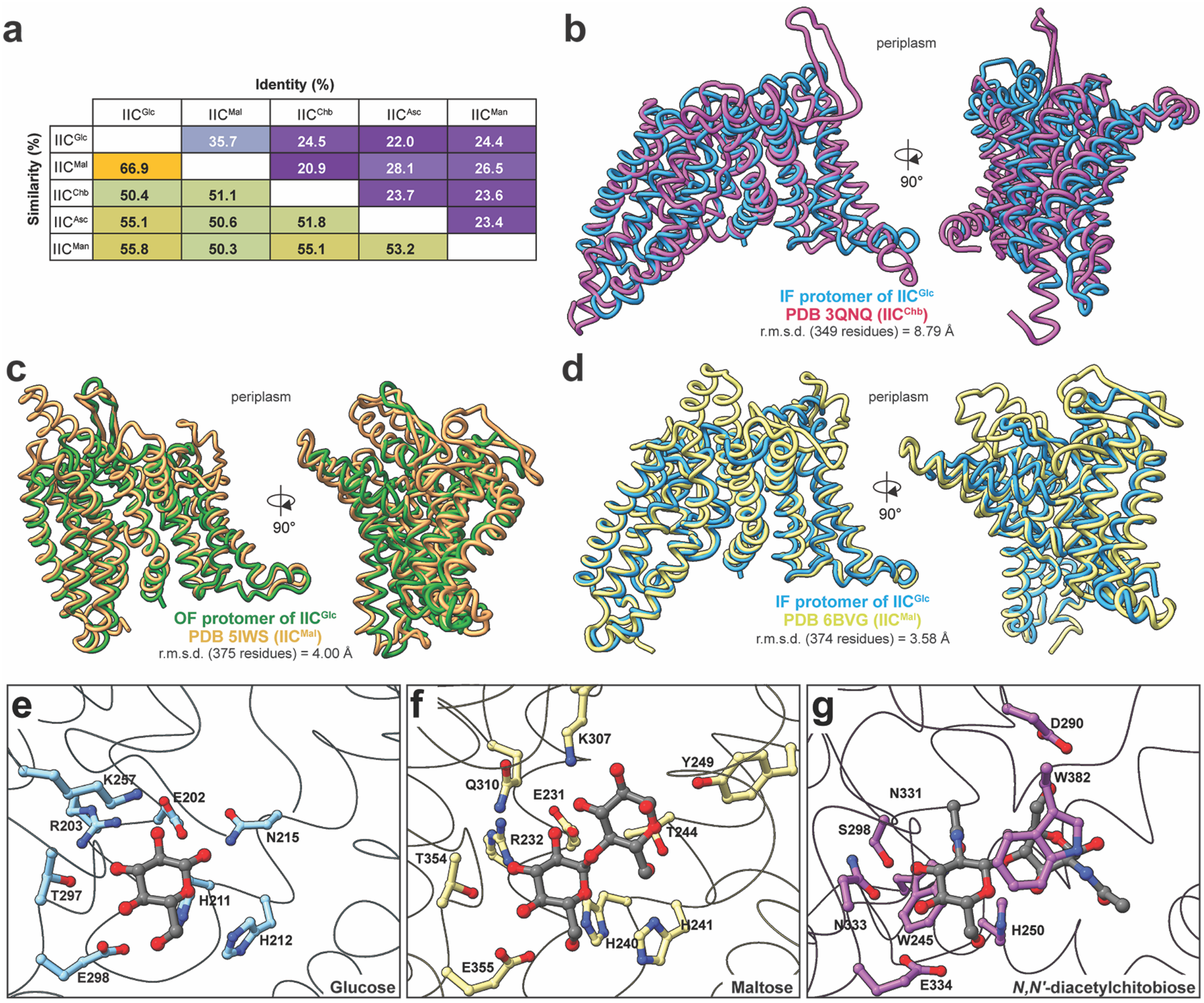

Similarity (%) \ Identity (%)	IIC^{Glc}	IIC^{Mal}	IIC^{Chb}	IIC^{Asc}	IIC^{Man}
IIC^{Glc}		35.7	24.5	22.0	24.4
IIC^{Mal}	66.9		20.9	28.1	26.5
IIC^{Chb}	50.4	51.1		23.7	23.6
IIC^{Asc}	55.1	50.6	51.8		23.4
IIC^{Man}	55.8	50.3	55.1	53.2	

Supplementary Fig. 4: Structural comparison of IIC^{Glc} with other IIC PTS transporters. (**a**) Amino acid sequence identity and similarity of selected IIC PTS transporters. Analysis was performed with LALIGN (https://www.ebi.ac.uk/jdispatcher/psa/lalign) using the following protein sequences (UniProtKB ID and range of considered sequence length is indicated in brackets): IIC^{Glc} (P69786, 1-388), IIC^{Mal} (P19642, 1-431), IIC^{Chb} (P17334, 1-452), IIC^{Asc} (P39301, 1-465) and IIC^{Man} (P69801, 1-266). Abbreviations used: Glc, Glucose; Mal: Maltose; Chb: *N,N′*-diacetylchitobiose; Asc: Ascorbate, Man: Mannose. (**b**), (**c**) and (**d**) depict the structural alignments of IIC^{Glc} with IIC^{Chb} and IIC^{Mal}, respectively. Protein Data Bank (PDB) IDs used for comparison with the structure of IIC^{Glc} in the IF (inward-facing) or OF (outward-facing) states are indicated along with the calculated r.m.s.d. (**e**), (**f**) and (**g**) illustrate a comparison of the ligand binding sites of IIC^{Glc} (glucose-bound), IIC^{Mal} (PDB identifiers: 5IWS and 6BVG, maltose-bound) and IIC^{Chb} (PDB identifier: 3QNQ, *N,N′*-diacetylchitobiose-bound), respectively. Structures were aligned relative to the glucose in IIC^{Glc}.

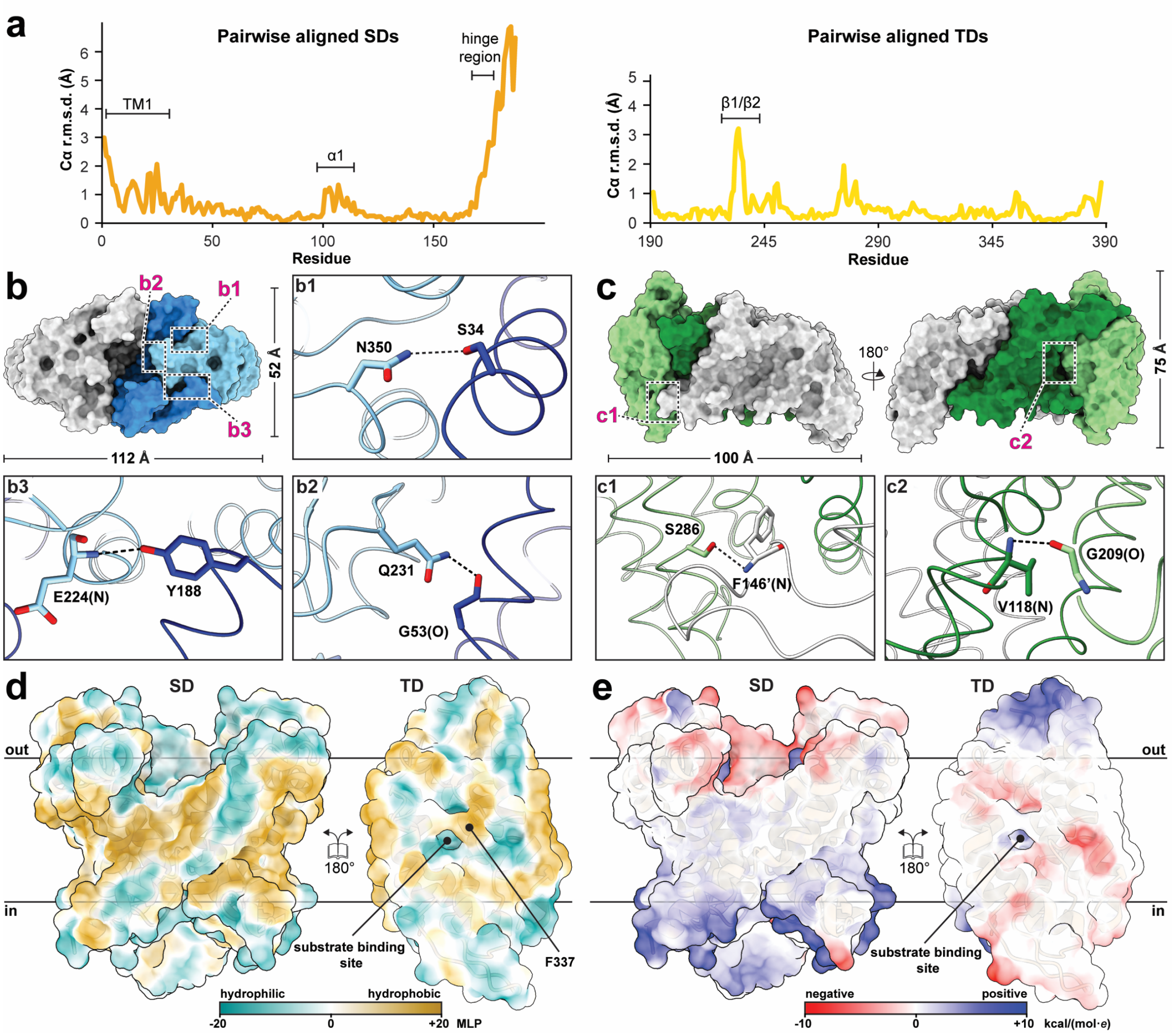

Supplementary Fig. 5: Analysis of scaffold (SD) and transport domain (TD) interactions. (**a**) Pairwise deviation plots of individually aligned SDs (*left*) and TDs (*right*) of inward-facing (IF) and outward-facing (OF) states highlight the conformational differences in specific structural elements. (**b**) and (**c**) map the transient interactions between the TD and SD in IF and OF states on surface representations of both states. Small panels provide zoom-in views on the hydrogen bond interactions. O and N in parentheses denote the backbone carbonyl oxygen and nitrogen atoms. Prime (‘) indicates residues from the opposite protomer. SDs are dark and TDs light blue/green, the other protomer is each in grey. (**d**) Hydrophobic surface rendering of the SD/TD interfaces (in the IF state) coloured according to the negative or positive molecular lipophilicity potential (MLP) either turquoise or brown, respectively. Note the hydrophobic patch (corresponding to F337) shielding the substrate binding site. (**e**) View on the electrostatic surface rendering (red and blue correspond to negative and positive electrostatic potential, indicated in kcal/(mol·*e*) of the opened SD/TD interface (in the IF state) as seen from the TD and SD, highlighting the complementary charged patches on the periplasmic and cytosolic sides.

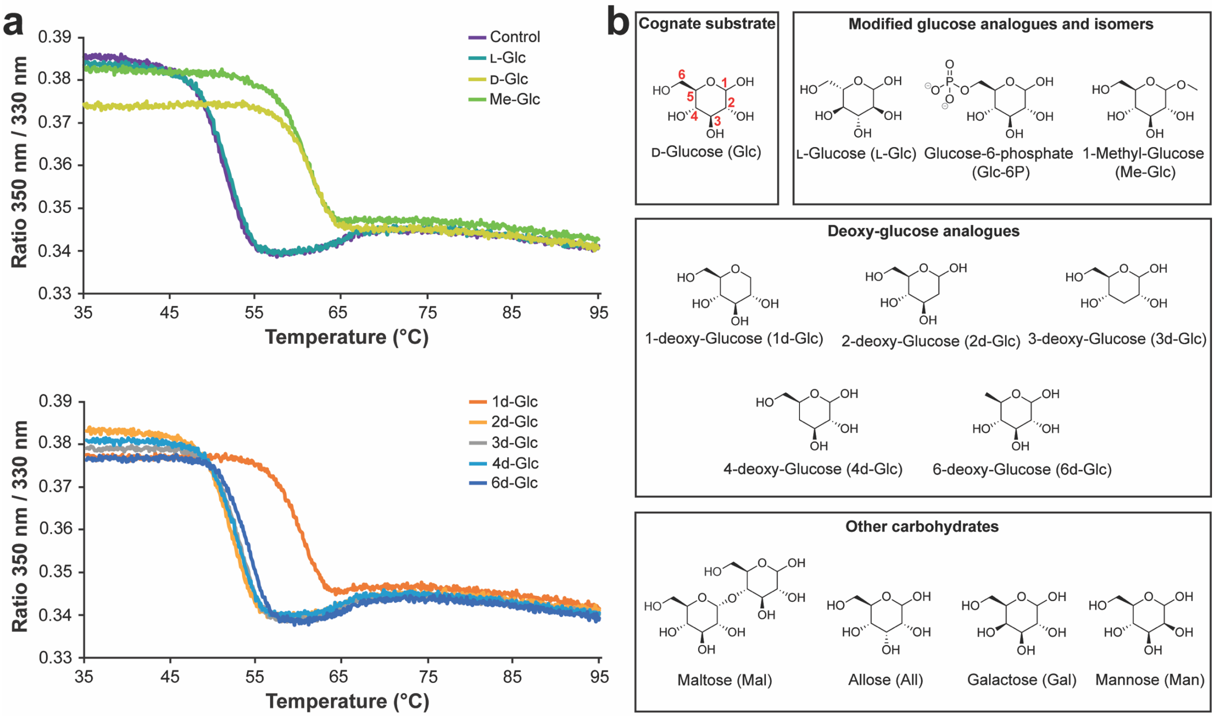

Supplementary Fig. 6: Label-free nano-differential scanning fluorimetry-based thermostability assay using different ligands. (**a**) Representative melting curves of purified $IICB^{Glc}$ in the presence of 10 mM of the indicated ligand or water (as control) (*above*), and deoxy-glucose analogues (*below*). (**b**) Overview and chemical structures and abbreviations of the different molecules used in this study. If not otherwise stated, D-isomers are meant.

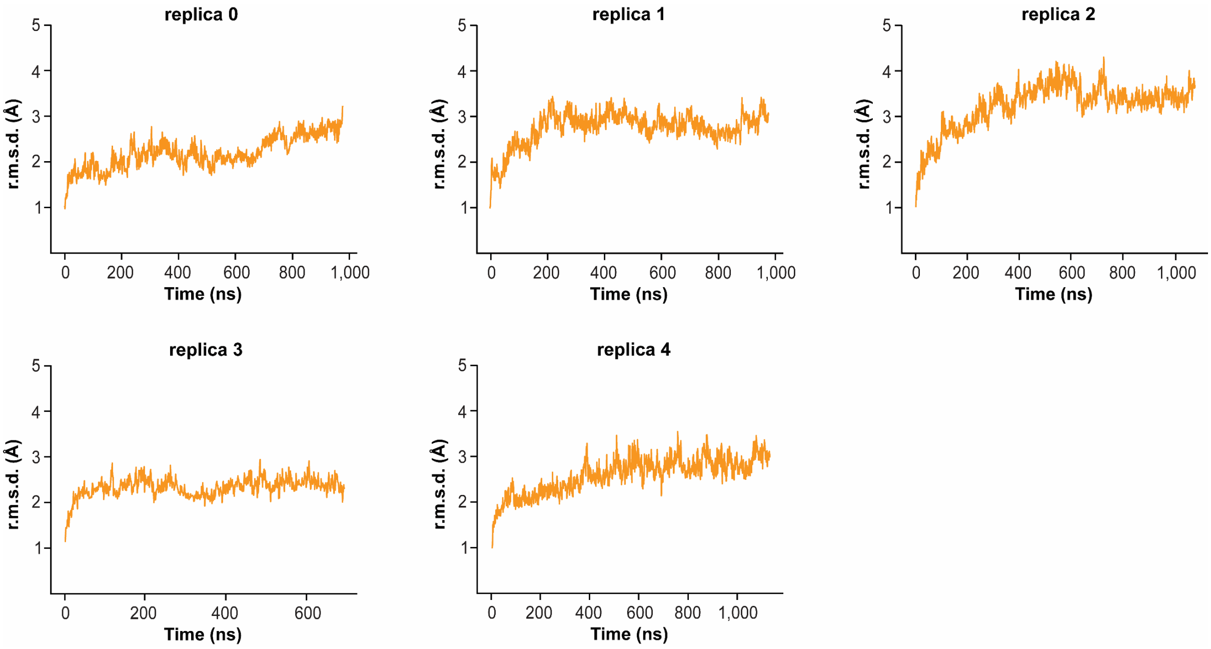

Supplementary Fig. 7: Root-mean-square deviation (r.m.s.d.) in Å across simulation replicas. The r.m.s.d. of all five replicas during the production phase are individually shown. Simulations were seeded from the lipid-bilayer embedded IF-OF conformation structure (PDB identifier: 8QST), as detailed in the Methods section.

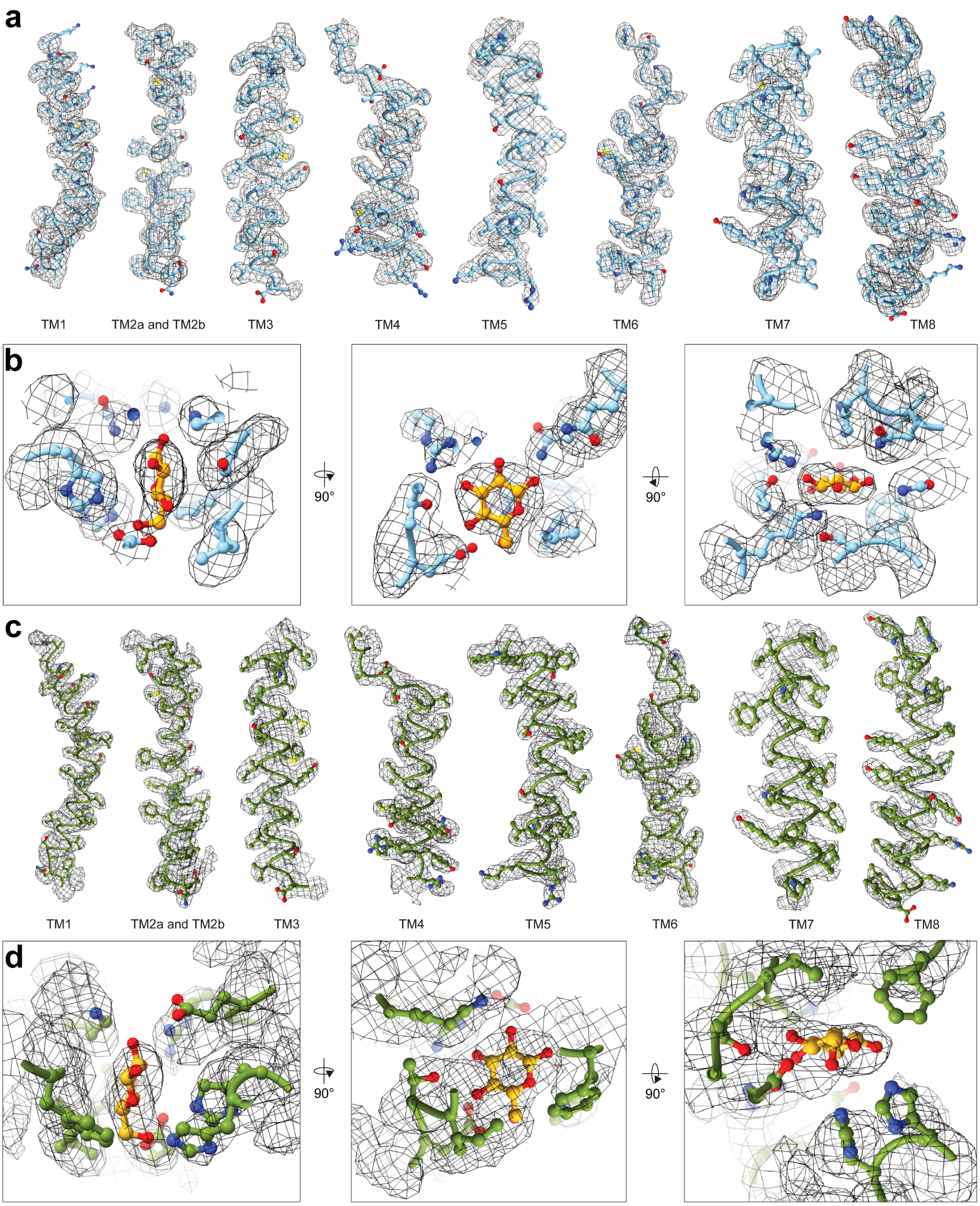

Supplementary Fig. 8: Cryo-EM map quality. (**a**) and (**c**) display the cryo-EM density maps with fitted models for transmembrane α-helices (TMs) for the inward- (IF; in blue) and outward-facing (OF; in green) conformation structures, respectively. (**b**) and (**d**) display the cryo-EM maps with fitted models for the D-glucose (in yellow) including the surrounding residues of the ligand binding site for the IF- and OF-facing conformation structures, respectively.

Supplementary Tables

Supplementary Table 1: Statistics for cryo-EM data collection, refinement and validation.

	**IICB^{Glc} IF-IF**<br>PDB 8QSR<br>EMD-18640	**IICB^{Glc} IF-OF**<br>PDB 8QST<br>EMD-18641
**Data collection and processing**		
Microscope, camera	Krios G2, K3	
Magnification	130,000	
Voltage (kV)	300	
Electron exposure ($e^{-}/Å^2$)	60.4	
Defocus range (μm)	-0.8 to -1.8	
Pixel size (Å)	0.645	
Micrographs	12,348	
Symmetry imposed	C_2	C_1
Final particle images (no.)	237,565	277,174
Map resolution (Å)	2.56	2.89
FSC threshold	0.143	0.143
**Refinement**		
Model resolution (Å)	2.6	3.0
FSC threshold	0.5	0.5
Model composition		
Non-hydrogen atoms	5,808	5,808
Protein residues	766	766
Ligands	2	2
B factors ($Å^2$)		
Protein	45.02	49.60
Ligand	51.30	56.87
Root-mean-square deviations (r.m.s.d.)		
Bond lengths (Å)	0.003	0.005
Bond angles (°)	0.512	0.580
Validation		
MolProbity score	1.21	2.10
Clashscore	4.34	10.9
Poor rotamers (%)	0.34	1.53
Ramachandran plot		
Favored (%)	98.16	93.96
Allowed (%)	1.84	5.78
Disallowed (%)	0.00	0.26

Abbreviations used: e^{-}, electron(s); FSC, Fourier-shell correlation; IF-IF, inward-facing - inward facing; IF-OF, inward-facing - outward-facing; kV, kilovolt

Supplementary Table 2: System set-up for MD simulations.

Starting structure (PDB ID)	Dimensions (Å)	Number of atoms	Number of water molecules	NaCl (mM)	Lipid composition	
					POPE	POPG
8QST	140×140×106	193,434	37,355	150	411	137

Abbreviations used: POPE, 1-palmitoyl-2-oleoyl-*sn*-glycero-3-phosphoethanolamine; POPG, 1-palmitoyl-2-oleoyl-*sn*-glycero-3-phosphoglycerol; ID, identifier
