# Supplementary material for: Structure and mechanism of a phosphotransferase system glucose transporter
Source: Nat Commun. 2024 Sep 12;15:7992. doi: 10.1038/s41467-024-52100-3 (PMC11393339; doi:10.1038/s41467-024-52100-3)
Supplement: Supplementary file 3 — Description of Additional Supplementary Files [file 41467_2024_52100_MOESM3_ESM.pdf]

Description of Additional Supplementary Files

File Name: Supplementary Movie 1

Description: Rigid body motion of the TD (yellow), resulting in a vertical shift of ~13 Å for the substrate binding site.

File Name: Supplementary Movie 2

Description: Glucose molecule enters the OF substrate binding pocket
