# Supplementary figures and images for: Structure and mechanism of a phosphotransferase system glucose transporter
Source: Nat Commun. 2024 Sep 12;15:7992. doi: 10.1038/s41467-024-52100-3 (PMC11393339; doi:10.1038/s41467-024-52100-3)

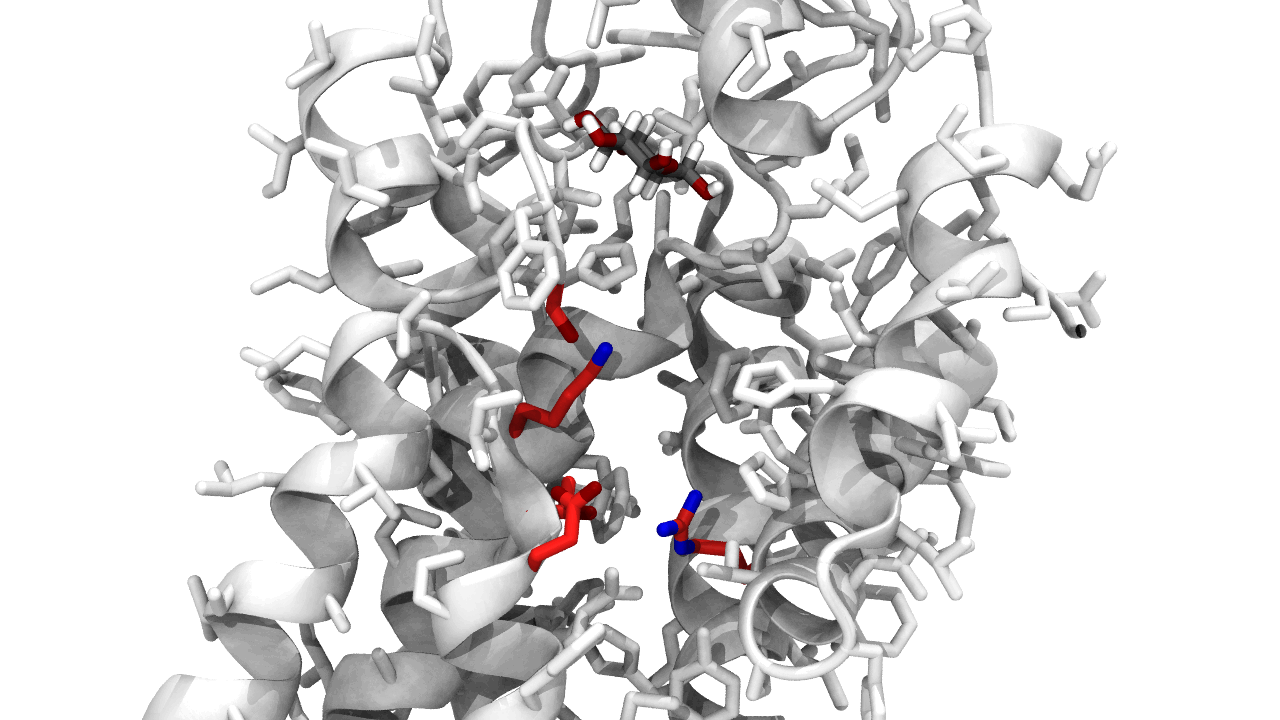

Supplement: Supplementary file 5 — Supplementary Movie 2 [file 41467_2024_52100_MOESM5_ESM.gif]
